# T Cell Responses Induced by Adenoviral Vectored Vaccines Can Be Adjuvanted by Fusion of Antigen to the Oligomerization Domain of C4b-Binding Protein

**DOI:** 10.1371/journal.pone.0044943

**Published:** 2012-09-12

**Authors:** Emily K. Forbes, Simone C. de Cassan, David Llewellyn, Sumi Biswas, Anna L. Goodman, Matthew G. Cottingham, Carole A. Long, Richard J. Pleass, Adrian V. S. Hill, Fergal Hill, Simon J. Draper

**Affiliations:** 1 The Jenner Institute, University of Oxford, Oxford, United Kingdom; 2 Laboratory of Malaria and Vector Research, National Institute of Allergy and Infectious Diseases/National Institutes of Health, Rockville, Maryland, United States of America; 3 Liverpool School of Tropical Medicine, Liverpool, United Kingdom; 4 Imaxio SA, Lyon, France; Federal University of São Paulo, Brazil

## Abstract

Viral vectored vaccines have been shown to induce both T cell and antibody responses in animals and humans. However, the induction of even higher level T cell responses may be crucial in achieving vaccine efficacy against difficult disease targets, especially in humans. Here we investigate the oligomerization domain of the α-chain of C4b-binding protein (C4 bp) as a candidate T cell “molecular adjuvant” when fused to malaria antigens expressed by human adenovirus serotype 5 (AdHu5) vectored vaccines in BALB/c mice. We demonstrate that i) C-terminal fusion of an oligomerization domain can enhance the quantity of antigen-specific CD4^+^ and CD8^+^ T cell responses induced in mice after only a single immunization of recombinant AdHu5, and that the T cells maintain similar functional cytokine profiles; ii) an adjuvant effect is observed for AdHu5 vectors expressing either the 42 kDa C-terminal domain of *Plasmodium yoelii* merozoite surface protein 1 (PyMSP1_42_) or the 83 kDa ectodomain of *P. falciparum* strain 3D7 apical membrane antigen 1 (PfAMA1), but not a candidate 128kDa *P. falciparum* MSP1 biallelic fusion antigen; iii) following two homologous immunizations of AdHu5 vaccines, antigen-specific T cell responses are further enhanced, however, in both BALB/c mice and New Zealand White rabbits no enhancement of functional antibody responses is observed; and iv) that the T cell adjuvant activity of C4 bp is not dependent on a functional Fc-receptor γ-chain in the host, but is associated with the oligomerization of small (<80 kDa) antigens expressed by recombinant AdHu5. The oligomerization domain of C4 bp can thus adjuvant T cell responses induced by AdHu5 vectors against selected antigens and its clinical utility as well as mechanism of action warrant further investigation.

## Introduction

The development of highly efficacious vaccines against difficult disease targets such as malaria, tuberculosis (TB), HIV-1 and pandemic influenza, will likely require the induction of strong cellular immunity as well as antibody responses. Whilst classical protein-in-adjuvant vaccines primarily induce antibodies, viral vectored vaccines are being developed as a means of inducing both T cell and antibody responses [1]. Encouragingly, new generation vaccine delivery platforms, primarily based on recombinant adenovirus and poxvirus prime-boost immunization regimes, are capable of inducing T cell responses in non-human primates [2]–[5] and humans [6]–[9] of a higher magnitude than ever reported before. However, the development of adjuvants to enhance the T cell responses induced by viral vectored vaccines may be crucial to the ultimate development of highly effective vaccines against such difficult infectious diseases and cancers.

The oligomerization domain of complement C4b-binding protein (C4 bp) α-chain has been identified as an adjuvant of antibody responses in both protein and viral vectored vaccination regimes [10], [11]. Full-length native C4 bp is an inhibitor of the classical and lectin pathways of complement activation. It consists of seven α-chains, one of which may be replaced by a β-chain in humans, linked together by a heptamerization domain at the C-terminus of the α-chains [12]. This oligomerization domain is 57 amino acid residues in humans and 54 amino acid residues in mice. It contains an amphipathic α-helix region, which is necessary and sufficient for heptamerization, as well as two cysteine residues which stabilize the structure [13]. Previous studies have shown that fusion of this domain to other proteins causes them to oligomerize [10], [14]–[16]. When the murine C4 bp oligomerization domain (henceforth known as IMX108 [10]) was fused to the 19 kDa C-terminal domain of *Plasmodium yoelii* merozoite surface protein 1 (PyMSP1_19_) recombinant protein it increased antigen-specific antibody responses following vaccination and enhanced protection against challenge with red blood cells infected with *P. yoelii* (pRBC) in mice [10]. Furthermore, when this domain is fused to PyMSP1_42_ in human adenovirus serotype 5 (AdHu5) and modified vaccinia virus Ankara (MVA) viral vectored vaccines and given in a prime-boost regime eight weeks apart (Ad-M42), then enhanced antibody responses are induced [11]. Although T cell responses were also apparently increased in that study, this finding appeared less consistent so here we have investigated T cell immunogenicity in detail using each vector alone with multiple blood-stage malaria antigens each fused to the oligomerization domain of C4 bp.

In a previously reported study, immunization of mice with murine C4 bp (PyMSP1_19_+IMX108 fusion protein) led to the induction of auto-antibodies against murine C4 bp [10]. This led the authors to test the C4 bp α-chain oligomerization domains from a variety of other mammalian and avian species for adjuvant activity in mice without induction of auto-antibodies [10]. All the C4 bp oligomerization domains tested formed soluble heptameric proteins, and induced higher antigen-specific antibody titers in mice than PyMSP1_19_ protein alone or in Freund’s adjuvant. The most immunogenic form, termed IMX313, is a hybrid derived from the oligomerization domains of the two chicken orthologues of C4 bp α-chain. It was designed to minimize similarity to mammalian C4 bp α-chain domains and has less than 20% amino acid identity to human C4 bp. The IMX313 sequence therefore minimizes the potential for auto-antibody induction in humans, and is a candidate “molecular adjuvant” for enhancement of vaccine-induced immune responses in humans. This construct has also recently been tested as a C-terminal fusion to the *Mycobacterium tuberculosis* antigen 85A in the clinical vaccine candidate MVA85A and significantly increased immune responses in both mice and rhesus macaques [17].

Surprisingly, in the recombinant protein vaccine study by Ogun *et al.*, the antibody titers induced against PyMSP1_19_ (when fused to different C4 bp α-chain oligomerization domain homologues) varied significantly, and the majority led to significantly higher antibody titers than a non-related heptameric oligomerization domain from *P. abyssi* [10]. It was therefore hypothesized that antigen oligomerization is not the sole mechanism explaining the adjuvant activity of this structural motif. The central core domain of C4 bp has been reported to contribute to binding CD40 on B cells [18], as well as to the pentraxins, serum amyloid P component (SAP) [19], [20] and C-reactive protein (CRP) [21]. We therefore hypothesized that adjuvant activity may occur due to binding of the oligomerization domain of C4 bp to these acute-phase proteins. These proteins can function by binding Fcγ receptors (FcγR) on leukoctyes, resulting in enhanced FcγR-mediated phagocytosis and cytokine secretion [22], which may consequently lead to an enhanced adaptive immune response. An alternative hypothesis is that oligomerization of the vaccine antigen may lead to formation of immune complexes following antibody induction, therefore enhancing activation via FcγR.

The studies presented here represent an iterative process of vaccine development. Initial studies show that C-terminal fusion of the murine oligomerization domain IMX108 to *P. yoelii* MSP1_42_ can increase the magnitude of the T cell response induced by adenoviral vectored vaccines in mice. Given these promising results, as well as our previously published results [11], we then tested the IMX313 variant, which is suitable for use in humans, fused to *P. falciparum* blood-stage antigen clinical constructs [6], [7], [23], [24]. Here we demonstrate that IMX313 can similarly increase the magnitude of T cell responses induced by adenoviral vectors in mice. We find that the observed adjuvant activity is associated with oligomerization of small (<80 kDa) antigens and is not dependent on a functional Fc-receptor γ-chain in the host**.**

## Results

### IMX108 Enhances T Cell, but not Antibody Responses, Following a Single Immunization with AdHu5-PyMSP1_42_

**Figure 1:**
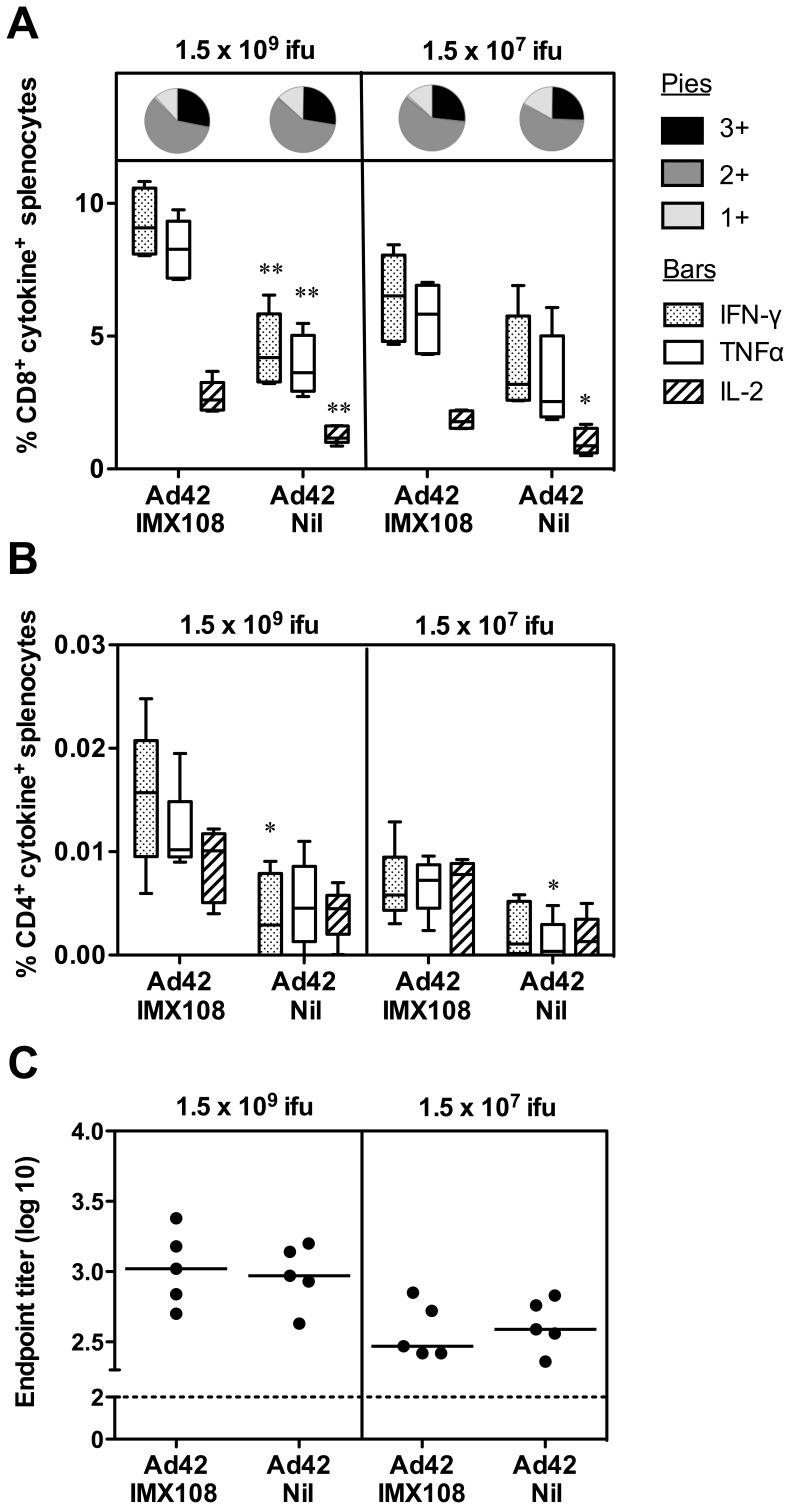
Adjuvant activity of IMX108 following immunization with AdHu5-PyMSP1_42_. BALB/c mice were immunized i.m. with 1.5×10^9^ or 1.5×10^7^ ifu AdHu5-PyMSP1_42_±IMX108 (Ad42-IMX108 or Ad42-Nil). Fourteen days post immunization splenocytes were re-stimulated with PyMSP1_33_ peptides and assessed for frequency of IFN-γ, IL-2, and TNFα positive CD8^+^ (A) and CD4^+^ (B) T cells by ICS. Box and whisker plots show median, IQR and range. The pie charts show the functional composition of the response and represent the proportion of PyMSP1_33_^−^specific CD8^+^ T cells positive for 1, 2 or all 3 cytokines measured. Fourteen days post-immunization total PyMSP1_19_-specific IgG responses were measured by ELISA (C). Data points represent individual mice and bars indicate median titer. n = 5 mice per group. **P<0.01, *P<0.05 compared to AdHu5-PyMSP1_42_+IMX108 at the same dose by Mann Whitney test. Data are representative of two independent experiments.

We have previously demonstrated that following priming with AdHu5 and boosting eight weeks later with MVA, both expressing PyMSP1_42_ fused to IMX108, antigen-specific IgG and CD4^+^IFN-γ^+^ responses are significantly increased compared to the same immunization regime with vectors expressing PyMSP1_42_ without an IMX108 fusion. CD8^+^IFN-γ^+^ responses also trended towards being higher, but were not significant, and enhanced protection against blood-stage *P. yoelii* challenge was observed [11]. In these experiments immunogenicity after a single vaccination was not assessed. A previous study adjuvanting an AdHu35 vector with aluminium phosphate (AdjuPhos) showed a clear dose sparing effect of the adjuvant [25], and thus in order to test whether IMX108 could enhance immunogenicity after a single immunization, we immunized BALB/c mice with two different doses of AdHu5-PyMSP1_42_ with or without IMX108 (hereafter known as Ad42-IMX108 or Ad42-Nil). Vaccines were given by the intramuscular route (i.m.) as this is the route used for clinical administration in our recent vaccine trials [6], [7]. Following a single immunization, the frequency of antigen-specific CD8^+^ T cell responses at the higher dose was significantly increased in terms of cells positive for IFN-γ, TNFα and IL-2 if the vaccine included IMX108 (Figure 1A). At the lower dose, only IL-2 was significantly increased if the vaccine included IMX108. IFN-γ and TNFα responses trended towards being higher with IMX108, but did not reach statistical significance (*P* = 0.0556 for both cytokines) (Figure 1A). This is consistent with the dose sparing effect observed by Ophorst *et al.* [25]. It has been suggested that the multi-functional “quality” of T cells in terms of the number of cytokines produced may be important for protection against *Leishmania major* [26] and liver-stage *P. berghei* malaria in mice [27]. Given an adjuvant may potentially change the functionality or phenotype of the T cells induced, we also analyzed cells for their ability to co-produce the three cytokines tested using intracellular cytokine staining (ICS) and flow cytometry. This revealed that the “quality” of the CD8^+^ T cell response in terms of the proportion of cells producing all three cytokines (3+, IFN-γ^+^TNFα^+^IL-2^+^), two cytokines (2+, predominantly IFN-γ^+^TNFα^+^IL-2^−^) or a single cytokine (1+, predominantly IFN-γ^+^TNFα^-^IL-2^−^) was unaffected by addition of IMX108 to the PyMSP1_42_ antigen (Figure 1A). A trend towards increased CD4^+^ responses was observed with IMX108 at both doses, with significantly greater production of IFN-γ at the high dose, and greater production of TNFα at the lower dose (Figure 1B). However, these CD4^+^ responses were too low for accurate multi-functional analysis (data not shown). Also, in contrast to the responses observed following an Ad-MVA prime-boost immunization regime [11], or a PyMSP1_19_ protein immunization [10], following a single Ad42 vaccination total PyMSP1_19_-specific IgG was not enhanced by IMX108 (Figure 1C).

Adenoviral vectors may be titered by two different methods, either by assessing infectious units (ifu) or by counting viral particles (vp). The measure of infectious units includes only antigen-producing viable virus, whilst vp also includes non-viably packaged DNA. We have found that ifu more accurately reflects immunogenicity [28], although clinically adenoviral vaccines are still administered based on dosing in vp [29]. We therefore also assessed whether the adjuvant effect was observed when mice were immunized based on vp. BALB/c mice were immunized i.m. with 10^10 ^vp or 10^8^ vp, or with 10^8^ vp i.d. AdHu5-PyMSP1_42_±IMX108 and a similar adjuvant effect for CD8^+^ T cell responses was observed (Figure S1). Furthermore, as expected the comparable responses were observed whether the vaccine was given by the i.m. or i.d. route (responses were not significantly different by two-way ANOVA for any cytokine tested). This is in agreement with previously published data in mice [30] and humans [8].

### Immunogenicity of AdHu5 Vaccines Expressing PfAMA1 and PfM128 with IMX313

**Figure 2:**
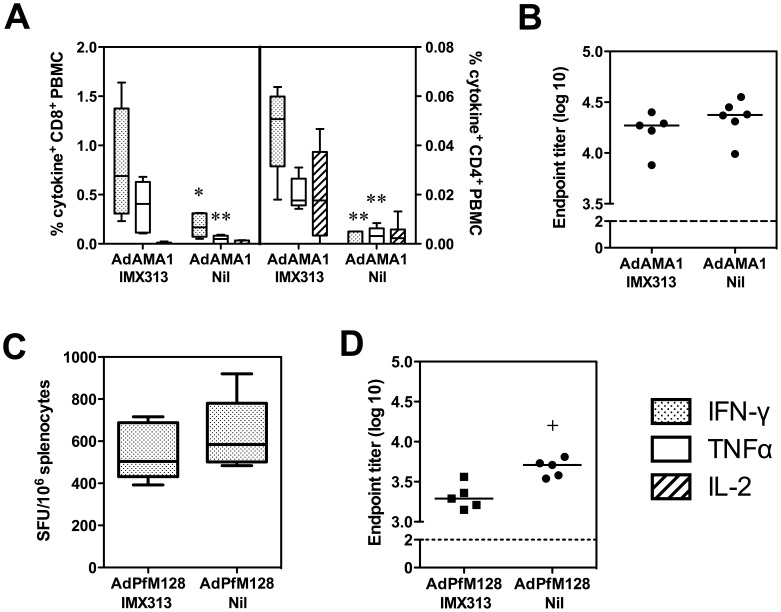
Adjuvant activity of IMX313 following immunization with AdHu5-PfAMA1 or AdHu5-PfM128. BALB/c mice were immunized i.m. with 1×10^9^ ifu AdHu5-PfAMA1±IMX313 (AdAMA1-IMX313 and AdAMA1-Nil). Twenty-one days post-immunization frequency of IFN-γ, TNFα and IL-2 positive CD8^+^ and CD4^+^ T cells was assessed in the blood by ICS following stimulation with PfAMA1 peptides (A). Antigen-specific total IgG was assessed by ELISA (B). Points represent individual mice and bars indicate median responses (n = 5–6 mice per group). **P<0.01, *P<0.05 compared to AdAMA1-IMX313 by Mann Whitney test. Data show the results of a single experiment. A similar result was observed by spleen IFN-γ ELISPOT (Figure S3). In a separate experiment, mice were immunized i.m. with 1×10^7^ ifu AdHu5-PfM128±IMX313 (AdPfM128-IMX313 and AdPfM128-Nil). Fourteen days later mice were culled and PfMSP1_33_-specific IFN-γ positive splenocytes were measured by ELISPOT (C). Data shown are spot forming units (SFU) per 10^6^ splenocytes. PfMSP1_19_-specific total IgG was assessed by ELISA (D). Bars indicate median responses (n = 5 mice per group). Box and whisker plots indicate median, IQR and range. The dashed line indicates the limit of detection. ^+^ *P*<0.05 compared to AdPfM128-IMX313 by Mann Whitney test.

A number of viral vectored vaccines encoding *P. falciparum* antigens are currently being tested in Phase I/IIa clinical trials in Oxford, UK [6]–[8] These include both adenoviral and MVA vectors expressing the *P. falciparum* antigens apical membrane antigen 1 (PfAMA1) [3], [24] and an optimized composite version of MSP1 (PfMSP1), termed PfM128 [23], [30]. The latter comprises a fusion of conserved blocks of PfMSP1 fused to both the Wellcome and 3D7 alleles of PfMSP1_42_ [23]. Given the enhanced T cell immunogenicity observed with AdHu5 and MVA vectors expressing the rodent malaria antigen PyMSP1_42_ with the murine form of the C4 bp oligomerization domain, IMX108, we investigated whether immunogenicity of these candidate *P. falciparum* antigens could be similarly enhanced. We generated AdHu5 vectors expressing each of these antigens fused to the hybrid chicken oligomerization domain, IMX313. This domain has been optimized for minimal sequence homology with human C4 bp α-chain oligomerization domain, is a candidate sequence for clinical development and has potent adjuvant activity when fused to PyMSP1_19_ protein [10] and the TB antigen 85A expressed by MVA [17]. When mice were immunized with AdHu5-PfAMA1 with or without IMX313 (AdAMA1-IMX313 and AdAMA1-Nil respectively), the frequency of antigen-specific IFN-γ and TNFα expressing CD8^+^ and CD4^+^ T cells in the blood of mice immunized with AdAMA1-IMX313 was significantly increased (in comparison to AdAMA1-Nil), as measured by ICS three weeks post-immunization (Figure 2A). Antigen-specific IL-2 positive CD4^+^ cells also showed a trend towards increased frequency, although this did not reach statistical significance (Figure 2A). In this experiment responses were measured in the blood, rather than the spleen, so that mice could be kept alive for further experiments. However, a similar result was observed when AMA1-specific responses in the spleen were measured by IFN-γ ELISPOT in an independent experiment (Figure S3). Antibody titers were also assayed by ELISA, and similar to the previous experiments with Ad42-IMX108, total antigen-specific IgG was not increased following immunization with a single dose of AdAMA1-IMX313 as compared to AdAMA1-Nil (Figure 2B).

In contrast to the results with PfAMA1 and PyMSP1_42_, after mice were immunized with AdHu5-PfM128 with or without IMX313 (AdPfM128-IMX313 or AdPfM128-Nil), MSP1-specific IFN-γ CD8^+^ T cell frequency in a spleen ELISPOT assay was not enhanced by the presence of IMX313 (Figure 2C). Furthermore, total PfMSP1_19_-specific IgG was of significantly lower titer if the vaccine contained IMX313 (*P* = 0.0032) (Figure 2D).

### Oligomerization of the Antigen *in vitro* may be Insufficient for *in vivo* Adjuvantation

**Figure 3:**
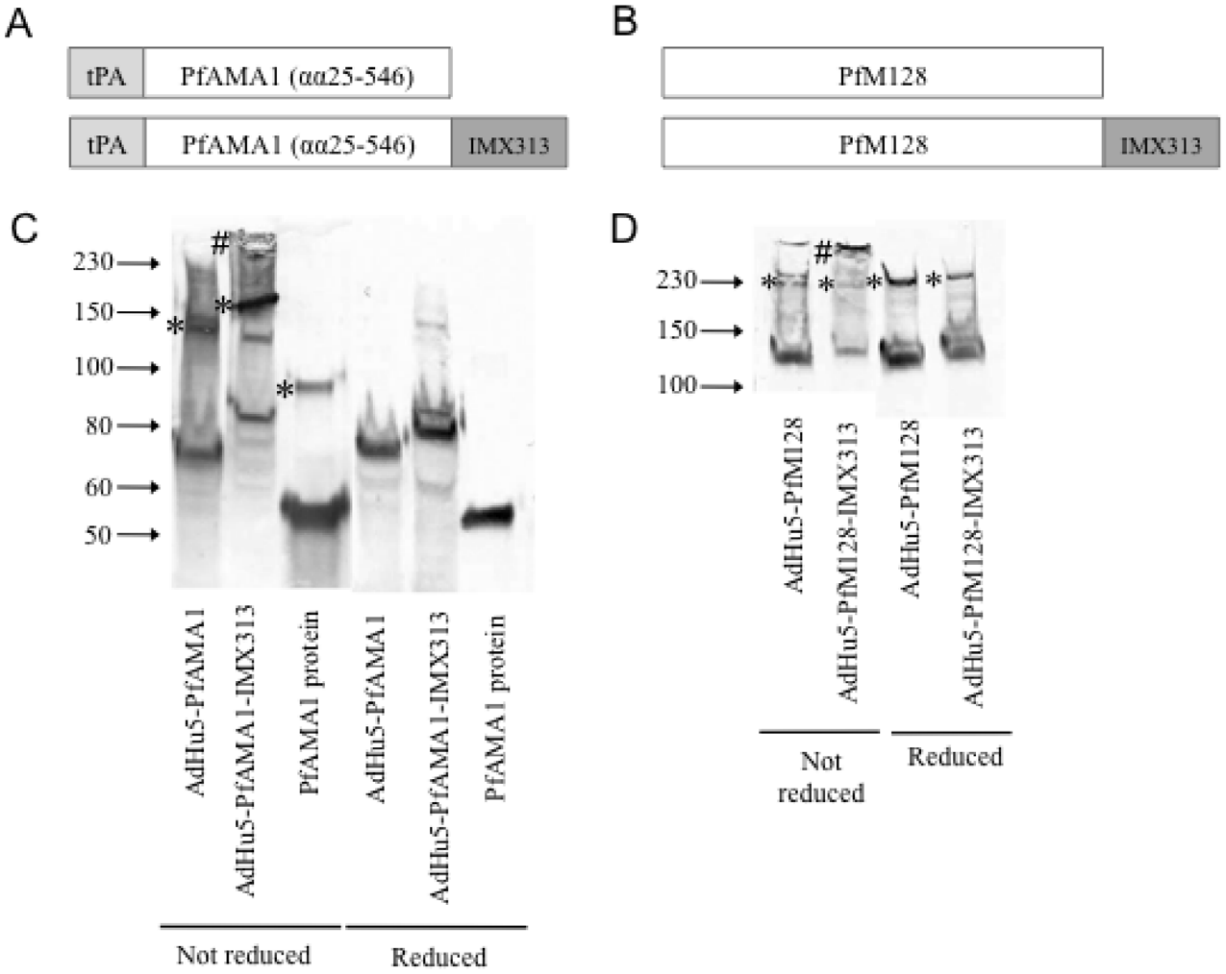
Schematic representation of PfAMA1±IMX313 and PfM128±IMX313 and assessment of antigen oligomerization by western blotting. Schematic representation of PfAMA1±IMX313 (A) and PfM128±IMX313 (B) constructs are shown; human tissue plasminogen activator signal peptide (tPA) (30 αα), PfAMA1 (521 αα), PfM128 (1111 αα) and IMX313 (55 αα). PfAMA1 3D7 protein was included as a positive control. Western blots probed with mouse anti-PfAMA1 polyclonal antibody (C) and mouse anti-PfMSP119 polyclonal antibody (D) under both reducing and non-reducing conditions. To the left of each column, * indicates presumed dimers and # indicates presumed multimers. Control AdHu5 infected lysate without the antigen of interest was also included and no bands were observed (data not shown).

Given that we observed a significant enhancement of T cell responses following AdHu5 vaccination with PyMSP1_42_ and PfAMA1 if the constructs included the oligomerization domain, but failed to see enhanced responses with PfM128-IMX313, we assessed whether PfAMA1 and PfM128 were indeed oligomerized by addition of the C4 bp domain. Western blotting revealed the presence of large molecular weight species, presumably heptamers, with both antigens (Figure 3). For un-fused PfAMA1 under reducing conditions monomers were observed. These were slightly faster migrating than the expected size of 83 kDa for the full ectodomain [31], and ran slower than the band observed with the 66 kDa PfAMA1 ectodomain recombinant protein, possibly indicating post-translational modification. The PfAMA1-IMX313 construct migrated slightly slower than PfAMA1 alone, indicating a higher molecular weight due to the addition of the IMX313 sequence. Under non-reducing conditions, larger bands were observed for all PfAMA1 proteins tested, presumably due to dimer formation. However, in the case of PfAMA1-IMX313, a much larger species was observed at the very top of the molecular weight range indicating that multimeric antigen was also present. A PfAMA1-IMX313 heptamer has an expected molecular weight of approximately 570 kDa. Although it is impossible to tell whether this species was precisely heptameric, these multimers were reduced upon addition of β-mercaptoethanol, in agreement with the known intermolecular disulphide bonds in this region of C4 bp [13]. These results are similar to those recently reported which show that IMX313 fused to the *M. tuberculosis* antigen 85A and delivered in an MVA vaccine induces enhanced T cell responses following antigen multimerization [17]. Interestingly, for the PfM128 antigen, a protein of the expected size (128 kDa) was observed in both reducing and non-reducing conditions. However, under non-reducing conditions this band was relatively weak and a large amount of protein in excess of 230 kDa was observed, again suggesting the presence of multimers. Under reducing conditions this large protein was no longer observed, and both monomers and dimers were present in greater quantities. These data therefore suggest that multimers are formed if the construct for both AdHu5-PfAMA1 and AdHu5-PfM128 includes IMX313. However, given the differences in adjuvantation seen for these two antigens, this property alone of the expressed antigen observed *in vitro* does not necessarily predict the ability of the motif to lead to enhanced T cell immunogenicity *in vivo*.

### Adjuvant Activity of IMX108/IMX313 in Homologous Prime-boost Regimes

**Figure 4:**
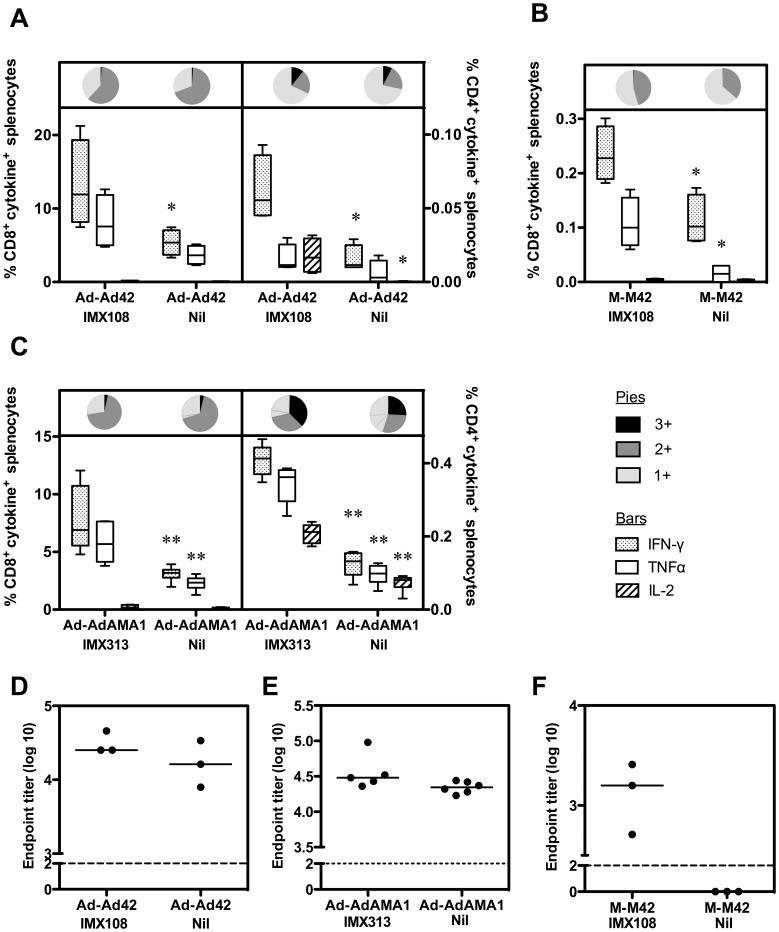
Adjuvant activity of IMX108/IMX313 in homologous prime-boost regimes. Frequency of antigen-specific IFN-γ, TNFα and IL-2 positive CD4^+^ and CD8^+^ T cells in the spleen were measured by ICS fourteen days post-boost following two immunizations with 5×10^10 ^vp AdHu5-PyMSP1_42_±IMX108 i.d. (Ad-Ad42+IMX108 and Ad-Ad42-Nil respectively) (A), 5×10^7^ pfu MVA-PyMSP1_42_±IMX108 i.d. (M-M42+IMX108 and M-M42-Nil) (B) or 1×10^9^ ifu AdHu5-PfAMA1±IMX313 i.m. (Ad-AdAMA1+IMX313 and Ad-AdAMA1-Nil) (C). Immunizations were given eight weeks apart. Box and whisker plots show median, IQR and range and pies show the proportion of cells positive for 1, 2 or all 3 cytokines. Antigen-specific total IgG responses were also measured in the serum fourteen days after boost by ELISA (D-F). Points represent individual mice and bars indicate median titer. The dashed line indicates the limit of detection. **P<0.01, *P<0.05 compared to the same vaccination regime with IMX108 or IMX313 by Mann Whitney test. Data show the results of single experiments.

Given the lack of adjuvant effect of IMX108 on IgG responses after a single immunization with AdHu5-PyMSP1_42_, but the observed adjuvant effect on IgG previously reported after an Ad-MVA prime-boost regime (Ad42-M42) [11], we investigated whether a homologous adenovirus boost could enhance IgG. Mice were immunized twice with AdHu5-PyMSP1_42_±IMX108, eight weeks apart (Ad42-Ad42). Fourteen days after the second immunization mice were culled and T cell and antibody responses assayed. Similar to a single immunization with Ad42 (Figure 1), or Ad42-M42 vaccination [11], CD4^+^ and CD8^+^ T cell responses trended towards increased frequency when IMX108 was fused to the antigen, with numbers of IFN-γ positive CD8^+^ and CD4^+^ T cells being significantly higher (Figure 4A). However, unlike prime-boost vaccination with Ad42-M42 [11], following Ad42-Ad42 immunization total PyMSP1_19_-specific IgG was not affected by addition of IMX108 (Figure 4D).

Similar experiments were carried out with AdHu5-PfAMA1. Mice were immunized with 1×10^9^ ifu AdHu5-PfAMA1±IMX313 i.m. twice (8 weeks apart) and T cell responses in the spleen were assayed by ICS two weeks after the boost. Overall, T cell responses were significantly higher than those observed following a single immunization with AdHu5-PfAMA1 (Figure 2A). Frequency of IFN-γ or TNFα positive CD8^+^ T cells was significantly higher when IMX313 was fused to PfAMA1, although IL-2 was almost undetectable even in the IMX313 group (Figure 4C). Production of all three cytokines from CD4^+^ T cells was significantly higher in the presence of IMX313. Again, however, antigen-specific total IgG was not enhanced by IMX313 (Figure 4E).

To investigate the role of MVA in enhanced antibody responses, mice were immunized twice with MVA-PyMSP1_42_±IMX108 eight weeks apart (M42-M42). Following the first immunization T cell and antibody responses were not detectable (data not shown). These data are consistent with the known poor suitability of MVA as a priming agent [1]. Following two immunizations, weak CD8^+^IFN-γ^+^ responses could be detected by ICS and were significantly higher if the vaccine included IMX108 (*P* = 0.029) (Figure 4B). Furthermore, PyMSP1_19_-specific IgG was detectable following vaccination with MVA expressing PyMSP1_42_ fused to IMX108, but not without (Figure 4F).

### Ad-AdAMA1-IMX313 does not Enhance Functional Antigen-specific IgG in Rabbits

**Figure 5:**
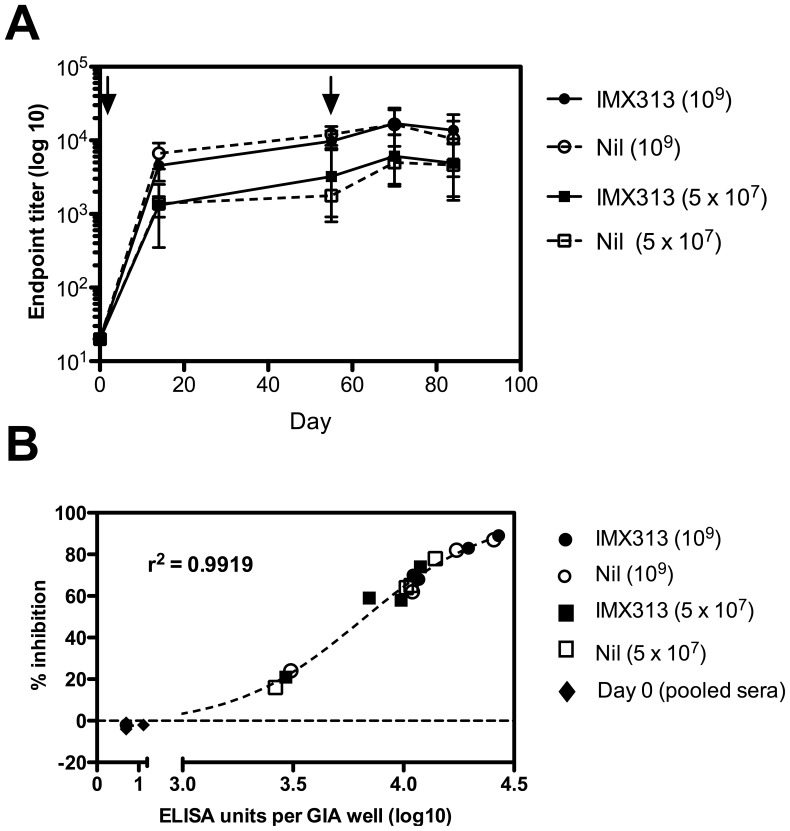
Adjuvanticity of IMX313 in rabbits following immunization with AdHu5-PfAMA1. New Zealand white rabbits were immunized i.m. with 1×10^9^ or 5×10^7^ ifu AdHu5-PfAMA1±IMX313, 56 days later they were given a homologous boost. Serum was taken on days 0, 14, 55, 70 and 84 and IgG titers were measured by ELISA (A). Median titer and range for n = 4 rabbits/group are shown. The arrows indicate the times of immunization. Purified IgG from day 84 post-immunization was assayed for GIA (B). Data presented are percentage inhibition for individual rabbits relative to the parasite growth observed without sera using purified IgG at 2.5 mg/mL. The non-linear regression curve is shown. Data show the results of a single experiment.

We also investigated whether IMX313 could enhance antibody responses in rabbits. New Zealand white rabbits were vaccinated i.m. with 1×10^9^ or 5×10^7^ ifu AdHu5-PfAMA1±IMX313 and boosted with the same vaccine 8 weeks later (Ad-AdAMA1-IMX313 or Ad-AdAMA1-Nil). The vaccines induced PfAMA1-specific IgG, but IMX313 did not significantly enhance antibody responses at any time-point at either the high or the low dose (Figure 5A), as was observed in mice. Purified IgG from sera taken on day 84 post priming immunization was tested for growth inhibitory activity (GIA) *in vitro* against *P. falciparum*. At the standard concentration of IgG used in the assay (2.5 mg/mL), the majority of rabbits showed moderate to high levels of GIA, with the exception of one rabbit in each of the low dose groups, and one rabbit that had the vaccine without IMX313 at the high dose (Figure 5B). Similar to previous studies [32], the GIA showed a sigmoidal relationship with ELISA titer of the purified IgG. However, there was no significant difference in the GIA of sera from rabbits that had been immunized with vaccines including IMX313 and those which had not. These data indicate that IMX313 altered neither the titer nor the functional activity of the antibodies induced. Higher levels of GIA were observed when IgG was used at 5 mg/mL in the assay, however, again no significant differences between vaccines was observed (data not shown).

### Adjuvant Activity of the C4 bp Oligomerization Domain is not Dependent on the FcR Common γ-chain

**Figure 6:**
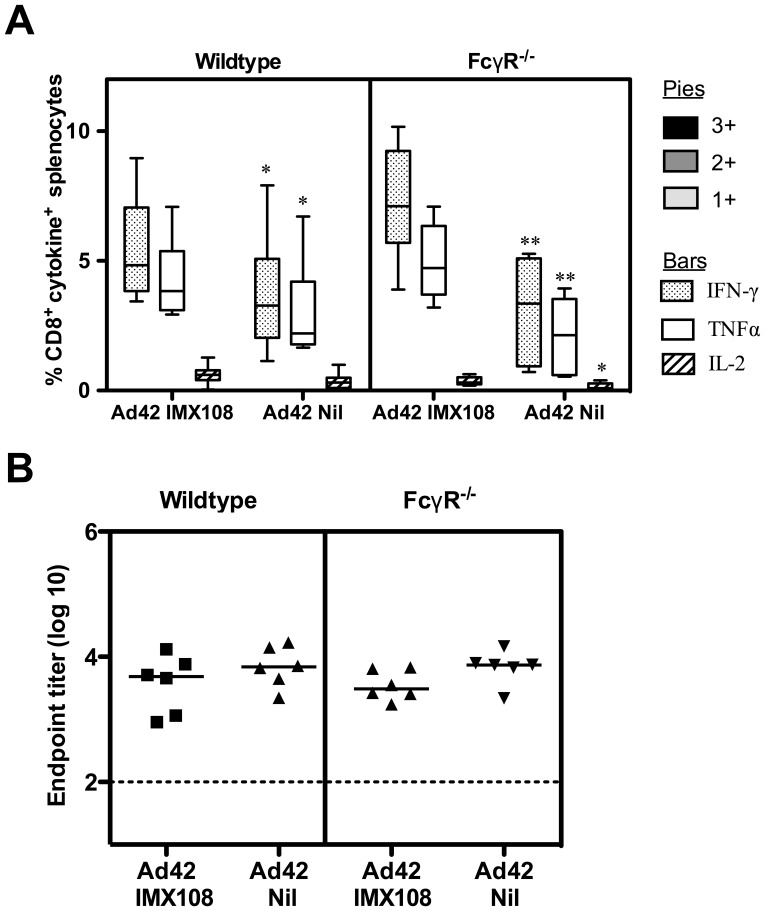
Adjuvanticity of IMX108 in FcRγ^−/−^ mice. Wildtype or FcRγ^−/−^ mice were immunized i.m. with 1.5×10^9^ ifu Ad42-IMX108 or Ad42-Nil. Two weeks later PyMSP1_33_-specific IFN-γ, TNFα and IL-2 positive CD8^+^ T cells in the spleen were measured by ICS (A). Box and whisker plots show median, IQR and range for n = 7–13 mice/group. PyMSP1_19_-specific total IgG was measured by ELISA (B). Points indicate individual mice and bars indicate median titer. n = 6 mice/group. *P<0.05, **P<0.01, by Mann Whitney test. Combined data are shown from two independent experiments.

Given the hypothesis that CRP and SAP binding to the oligomerization domain and then to FcγR may be the mechanism of IMX108/313 adjuvant activity, we investigated whether an adjuvant effect could still be observed in mice that were deficient in the FcR γ-chain (FcRγ^−/−^) which is common to the FcγRI, FcγRIII, FcγRIV and FcεRI [33], [34] in mice. FcRγ^−/−^ mice were immunized with AdHu5-PyMSP1_42_±IMX108. Two weeks later the mice were culled and PyMSP1_33_-specific CD8^+^ T cell responses in the blood were assayed by ICS and anti PyMSP1_19_-specific IgG responses were measured by ELISA. This showed that enhanced T cell responses due to IMX108 also occurred in the FcR common γ-chain knockout mice (Figure 6A), but antibody responses, like in wild-type mice, remained comparable (Figure 6B). These data indicate that FcγR that utilize the common γ-chain do not play a role in the T cell adjuvant activity of the C4 bp oligomerization domain. The knockout also did not appear to affect overall immunogenicity of the vaccination regimen, suggesting that the FcR γ-chain is not involved in priming immune responses induced by adenoviral vectored vaccines.

## Discussion

These data show that the C4 bp α-chain heptamerization domain can effectively adjuvant antigen-specific T cell responses to some, but not all, antigens when fused to their C-termini and delivered by an adenoviral vectored vaccine. Fusion of the domain led to enhanced production of antigen-specific IFN-γ, TNFα and IL-2 from both CD8^+^ and CD4^+^ cells following vaccination with AdHu5 vectors expressing PyMSP1_42_ or PfAMA1. The adjuvant did not affect the multi-functional quality of the CD8^+^ T cells induced in terms of the cytokines assessed by multi-parameter flow cytometry, but rather increased the frequency of cells positive for all three cytokines proportionately. These data are in contrast to some classical adjuvants, which skew responses towards production of Th1- or Th2-type cytokines [35].

This adjuvant activity is likely not solely dependent upon oligomerization of the antigen as no *in vivo* adjuvant effect was observed with the PfM128-IMX313 construct. However, this possibility cannot be ruled out because the PfM128-IMX313 western blot did not clearly identify whether this construct induces oligomers efficiently. One possible explanation of the failure of IMX313 to adjuvant T cells against the PfM128 antigen, may be that adjuvanticity requires not only oligomerization of the antigen, but also efficient extracellular secretion of such oligomeric complexes. Western blots were carried out using cell lysate and did not assess whether the antigens were secreted. PfM128-IMX313 is 1165 amino acids (αα) in length, whilst PyMSP1_42_^_^IMX108 is only 447 αα and PfAMA1-IMX313 is 624 αα. The large size of this protein in multimeric form may prevent it being efficiently secreted. Furthermore the antigen comprises a non-physiological fusion of blocks 1, 3, 5, 12, 16 and 17 of PfMSP1, which may impair secretion. Biswas *et al*. have shown that biallelic AMA1 expressed by adenoviral vectors is secreted [24]. Given that both cellular and humoral immune responses to this construct were lower than those observed with PfM128-Nil, it is possible that increasing the size of this large antigen even further was more detrimental, than beneficial, to efficient transgene expression. However, the finding that adjuvant activity did not occur for PfM128-IMX313, despite intracellular oligomerization of the antigen, does not exclude the possibility that oligomerization is a key factor in the adjuvanticity of this structural motif, provided that the protein is sufficiently secreted.

Due to the iterative nature of this study, in which the murine variant, IMX108, was tested with a *P. yoelii* antigen and then the variant suitable for use in humans, IMX313, was tested with *P.* falciparum antigens, a direct comparison between the adjuvant activity of IMX108 and IMX313 for a single antigen was not made here. However, the varying adjuvant activity observed with the C4 bp α-chain oligomerization domain from different species when fused to the PyMSP1_19_ protein vaccine, or an unrelated oligomerization domain [10], could be due to the variable stability and strength of oligomers formed [36].

We hypothesized that adjuvant activity may occur due to the oligomerization domain binding to CRP and SAP, which then bind to FcγR, resulting in enhanced innate and adaptive immunity. Whilst our data indicate that the FcR common γ-chain is not required for adjuvant activity, it remains to be investigated whether antigens fused to C4 bp and expressed by the viral vectors bind to CRP and SAP at all, and therefore their possible role in adjuvant activity cannot be excluded. Further studies will need to be carried out to determine whether binding of IMX313 to these soluble pentraxins occurs, in agreement with published data for the full-length C4 bp molecule [19]–[21], and whether this is important in adjuvant activity.

Particulate antigens, such as oligomerized proteins, are known to be a better target for both cross-presentation [37], [38] and phagocytosis and this may potentially explain the ability of these domains to enhance antigen-specific T cell responses following both MVA and AdHu5 expression. However, greater uptake by phagocytosis might be expected to enhance antibody (as well as T cell) responses, but surprisingly, following a single or double AdHu5 vaccination, no differences in total IgG induction were observed for any antigen when fused to the oligomerization domain either in BALB/c mice or rabbits. However, enhanced antigen-specific antibody responses were observed in mice following MVA-MVA PyMSP1_42_, and have been reported previously following Ad-MVA [11] or protein [10] immunization. This suggests that administration of MVA or protein is necessary for enhanced antibody responses following immunization with a vaccine containing IMX108 or IMX313. Whilst protein vaccines administered as heptameric particles may be more suited for antibody induction, differences in cellular tropism and/or innate immune responses to MVA versus AdHu5 may explain the ability of the oligomerized antigen to better stimulate B cell responses in the context of poxviral expression. These differences remain to be further investigated. In future studies it would also be interesting to compare boosting with IMX313 conjugated protein vaccines and MVA vaccines. We have extensively tested the utility of adenoviral-prime protein-boost regimes in mice and rhesus macaques [3], [30], [39], and a Phase Ia clinical trial of this approach is currently underway with the PfAMA1 antigen (ClinicalTrials.gov, Identifier NCT01351948). Although we have shown protein vaccines can boost T cell responses in mice, in head-to-head comparisons MVA is superior at boosting T cell responses in the absence of IMX313 [30], [39].

Overall, these data demonstrate that C4 bp α-chain oligomerization domains can effectively enhance antigen-specific CD8^+^ T cell responses induced by AdHu5 vectored vaccines expressing certain antigens, and that this effect is unaltered in FcR γ-chain knockouts. CD4^+^ T cell responses were low in this study and were only marginally increased by the addition of the oligomerization domain to the vaccine construct. We have not investigated the causes of antigen-dependence, but speculate that this adjuvant activity requires not only oligomerization of the antigen, but also its efficient secretion. Few adjuvants are known to significantly enhance T cell responses from viral vectored vaccines – a technology that is fast becoming a leading and clinically-relevant subunit vaccine delivery platform [1]. Given that the maximum achievable T cell responses are likely required to provide protective efficacy against cancers and difficult infectious diseases, IMX313, which has been optimized for use in humans, remains a strong candidate for assessment in T cell-inducing vaccine antigen constructs.

## Materials and Methods

### Ethics Statement

Animal procedures were carried out according to the UK Animals (Scientific Procedures) Act 1986 and were approved by the University of Oxford Animal Care and Ethical Review Committee (PPL 30/2414).

### Recombinant AdHu5 and MVA Vaccines

AdHu5 and MVA vectors expressing PyMSP1_42_ with or without IMX108 (Ad42-IMX108 and Ad42-Nil) have been previously described [11]. AdHu5 and MVA vectors expressing the *P.* *falciparum* AMA1 (3D7 strain) antigen (AdAMA1-Nil) and *P. falciparum* MSP1 (PfM128) antigen (AdPfM128-Nil) have also been described elsewhere [23], [24], [30]. Vectors expressing these *P.* *falciparum* antigens fused at the C-terminus to the 55 amino acid IMX313 motif (PfAMA1-IMX313 and PfM128-IMX313) were made by similar methods. Adenoviruses were titered for both the number of viral particles (vp) by UV spectrophotometry [11] and the number of infectious units (ifu) by immunostaining. Immunostaining was carried out by infection of T-REx™-293 cells (Invitrogen) with serial dilutions of virus. Forty-eight hours post infection cells were stained with anti-hexon antibody and detected with HRP-conjugated secondary antibody (Cambridge Bioscience).

### Animals and Immunizations

All mouse experiments were carried out in specific pathogen-free conditions. 6–8 week old female BALB/c mice were obtained from Harlan, UK, or Charles River Laboratories, UK. Fc-receptor common γ-chain deficient mice [40], [41] on a BALB/c background (FcRγ^−/−^) were provided from the Queen’s Medical Centre, Nottingham, UK and bred at the Wellcome Trust Centre for Human Genetics, Oxford, UK. Genotype was confirmed using DNA extracted from tissue samples and the Expand High Fidelity PCR Kit (Roche Diagnostics, UK) and previously described reaction conditions and primers (http://jaxmice.jax.org/strain/002847.html) except that a final concentration of 4 mM MgCl_2_ was used. Experiments in New Zealand white rabbits were carried out at Spring Valley Laboratories, USA.

All vaccines were formulated in endotoxin free PBS and given intramuscularly (i.m.) or intradermally (i.d.) in a total volume of 50 µL (25 µL/leg or ear) to mice and 100 µL (50 µL/leg) to rabbits. There was an eight-week interval between prime and boost vaccinations.

### Isolation of Cells

Mouse spleens were mechanically homogenized in PBS, passed through a 70 µm cell strainer and erythrocytes were removed by re-suspension of cells in ACK lysis buffer for 5 min. The reaction was stopped with PBS, then cells were centrifuged and re-suspended in complete MEM (10% Foetal Calf Serum, Pen/Strep, L-glutamine and 2-Mercaptoethanol). For isolation of PBMC, 7–10 drops of blood were collected from the tail vein into 200 µL 10 mM EDTA in PBS. Erythrocytes were removed by incubation with ACK lysis buffer and cells were re-suspended in complete MEM.

### Peptides

PyMSP1 responses were measured using a pool of 55 peptides spanning the entire length of PyMSP1_33_ [11], [42], [43]. These were each 15 amino acids (αα) long and overlapped by 10αα. PfAMA1 responses were measured following stimulation with a pool of three PfAMA1 peptides, which include one H2^d^ CD8^+^ T cell (KYVKNLDELTLCSRH) and two H-2^d^ CD4^+^ T cell epitopes (VFGKGIIIENSKTTF and NKKIIAPRIFISDDK). Antigen-specific responses against *P. falciparum* MSP1_33_ (Wellcome strain) following PfM128 immunization were measured using the two overlapping peptides encoding a known H-2^d^ CD8^+^ T cell epitope (NKEKRDKFLSSYNYI and DKFLSSYNYIKDSID) [23].

### Multi-parameter Flow Cytometry

Splenocytes or PBMC were incubated for 5 h at 37°C in the presence of 1∶1000 GolgiPlug (Brefeldin A) and 1 µg/mL of each peptide or peptide pool. Samples were then stored at 4°C overnight. Cells were centrifuged, re-suspended in 1∶100 Fc-block (anti-CD32/CD16) and incubated for 15 min, washed once in PBS and then incubated for 30 min with 1∶100 anti-CD4 PacificBlue or APC-Alexa750 (Clone GK1.5) and anti-CD8 PerCP-Cy5.5 or PacificBlue (Clone 53–6.7) in PBS. Cells were then washed twice in PBS and then permeabilized by incubation with Cytofix/Cytoperm for 15 min. The reaction was stopped by addition of PermWash, cells were washed in PermWash and then incubated for 30 min with anti-IFN-γ APC or PE (clone XMG1.2), anti-TNFα FITC or APC (clone MP6-XP22) and anti-IL-2 PE or FITC (clone JES6–54), all diluted 1∶100 in PermWash. Finally cells were washed twice in PermWash then re-suspended in PBS and samples were run on a CyAN ADP flow cytometer (Dako, UK). Data were analyzed using Flowjo v8.8.7. The gating strategy used is shown in Figure S2. For multi-functional analysis, boolean gates were used and data presented using Pestle v1.6.2 and SPICE v 5.1 [44]. Background responses in un-stimulated cells were subtracted from stimulated responses prior to analysis.

### *Ex vivo* IFN-γ ELISPOT

ELISPOT assays specific for IFN-γ were carried out as previously described [45] using coating and detecting antibodies from Mabtech. Briefly, plates were coated with anti-IFN-γ antibodies overnight, plates were then washed and blocked before addition of splenocytes and the indicated peptides at 1 µg/mL and incubation for 18–20 h at 37°C. Plates were then washed and responses detected by addition of a biotin-conjugated rat anti-mouse IFN-γ antibody, before addition of Streptavidin Alkaline Phosphatase polymer and development with Biorad AP conjugate development buffer. Cells were plated in duplicate and the mean response in spot forming units (SFU) per 10^6^ PBMC was calculated. Responses from un-stimulated control wells were subtracted.

### ELISA

Mouse total IgG was measured by ELISA as previously described [11]. 96-well Nunc-immuno Maxisorp plates were coated with PyMSP1_19_-GST fusion protein [11], recombinant 3D7 PfAMA1 protein (a gift from Dr Chetan Chitnis (ICGEB, New Delhi, India)) [3], [24] or 3D7 PfMSP1_19_-GST fusion protein [23] at a concentration of 2 µg/mL in PBS. All serum samples were diluted 1∶100 in PBS containing 0.05% Tween and then diluted three-fold down the plate. Optical density was read at 405 nm (OD_405_) and endpoint titer calculated as the x-axis intercept of the dilution curve at an absorbance value 3 SD greater than the OD_405_ for naïve mouse serum. Samples were tested in duplicate and a reference control serum was included in all assays. Rabbit IgG ELISAs were carried out using a standardized protocol at the GIA reference center (LMVR, NIAID, NIH) using 3D7 PfAMA1 antigen provided by David Narum at the Laboratory of Malaria Immunology and Vaccinology, NIH, USA. A standard curve was generated using reference sera run in duplicate on every plate and one ELISA unit is the reciprocal dilution required to give an O.D. of 1 in the standardized assay [32].

### GIA

Polyclonal rabbit IgG was purified from serum samples by Protein G affinity chromatography and adjusted to a concentration of 10 mg/mL in incomplete RPMI. A standardized assay of *in vitro* growth inhibitory activity (GIA) was carried out at the GIA reference center (LMVR, NIAID, NIH) as previously described [32]. Rabbit IgG from individual animals were tested at a final concentration of 5 mg/mL and 2.5 mg/mL against 3D7 clone *P.* *falciparum* parasites.

### Western Blotting

Cell lysate was prepared by infection of 293 cells with the indicated AdHu5 vaccines at an MOI of 5 infectious units. Thirty hours post infection, the media was removed, cells were washed once with PBS, scraped, centrifuged and re-suspended in non-reducing or reducing Laemmli’s sample buffer. Cells were then heated at 95°C for 5 min and sonicated for 1 min before storage at −20°C until use. For Western blotting, samples were separated by SDS-PAGE on an 8–16% polyacrylamide pre-cast gel (Pierce) in Tris-HEPES-SDS running buffer (100 mM Tris, 100 mM HEPES, 1% SDS, pH 8.0). Proteins were blotted onto a nitrocellulose membrane (Biorad) by electrophoretic transfer. The membrane was then washed twice in PBS, blocked for 1 h with 3% BSA/PBS, washed again, incubated for 1 h in antigen-specific primary antibody (Ab) diluted 1∶1000 in 3% BSA/PBS, washed, incubated for 1 h with donkey anti-mouse-alkaline phosphatase diluted 1∶3000, washed, and finally developed with BCIP/NBT solution (Sigma). Colorplus prestained protein ladder, broad range (10–230 kDa) (NEB), was included to determine the size of the proteins. Control proteins and non-recombinant AdHu5 infected lysate were included on each gel to confirm that primary Ab was antigen specific. Antibody for detecting PfM128 antigen was polyclonal sera from mice immunized three times with recombinant PfMSP1_19_ protein [39] and for detecting PfAMA1 antigen polyclonal sera was used from mice immunized with viral vectored vaccines (ChAd63-MVA) encoding PfAMA1 [24].

### Statistics

Data were analyzed using GraphPad Prism v5.01. The normality of the data was determined using the Kolmogorov-Smirnov one-sample test. For non-parametric data, two groups were compared using a Mann-Whitney test and data are presented as median with individual data points, or box and whisker plots representing median, interquartile range (IQR) and range. ELISA titers were logarithmically (log 10) transformed in order to normalize the data and allow parametric analysis. In all cases *P*<0.05 was considered significant (*P<0.05, **P<0.01 and ***P<0.001).

## Supporting Information

Figure S1 **Adjuvant activity of IMX108 following AdHu5 vaccine dosing based on viral particles (vp).** BALB/c mice were immunized with 1×10^10^ vp or 1×10^8^ vp Ad42-IMX108 or Ad42-Nil by either the i.m. or i.d. route as indicated. Two weeks later frequency of PyMSP1_33_-specific IFN-γ, TNFα and IL-2 positive CD8^+^ splenic T cells was measured by ICS. Box and whisker plots show median, IQR and range for n = 5 mice/group. **P<0.01, *P<0.05 by Mann Whitney test compared to the Ad42-IMX108 given by the same dose and route.
(TIFF)

Figure S2 **Representative ICS gating strategy.** All cellular marker and cytokine gates were drawn with forward scatter (FS (lin)) on the x axis. Cell clumps, doublets and triplets were excluded by gating on single cells in a plot showing FS (area) against FS (lin). Lymphocytes were then selected on FS against side scatter (SS (lin)). CD8^+^ cells were selected by first selecting CD4^−^ cells to ensure that CD4^+^CD8^+^ cells were excluded. Within the CD4^−^ cells, CD8^+^ cells were selected. Cytokine gates were then drawn for the CD8^+^ cells as shown and background from unstimulated cells subtracted. A similar gating strategy (including a CD8^−^ gate) was used for selecting CD4^+^ cells.
(EPS)

Figure S3 **Adjuvant activity of IMX313 following Ad-AMA1 immunisation assessed by IFN-γ ELISPOT.** BALB/c mice were immunized i.m. with 1×10^7^ ifu AdHu5-PfAMA1 with or without IMX313 (AdAMA1-IMX313 and AdAMA1-Nil). Fourteen days later mice were culled and AMA1-specific IFN-γ positive splenocytes were measured by ELISPOT. Data shown are spot forming units (SFU) per 10^6^ splenocytes (n = 5 mice per group). Box and whisker plots indicate median, IQR and range.
(TIFF)
