# Supplementary figures and images for: T Cell Responses Induced by Adenoviral Vectored Vaccines Can Be Adjuvanted by Fusion of Antigen to the Oligomerization Domain of C4b-Binding Protein
Source: PLoS One. 2012 Sep 12;7(9):e44943. doi: 10.1371/journal.pone.0044943 (PMC3440343; doi:10.1371/journal.pone.0044943)

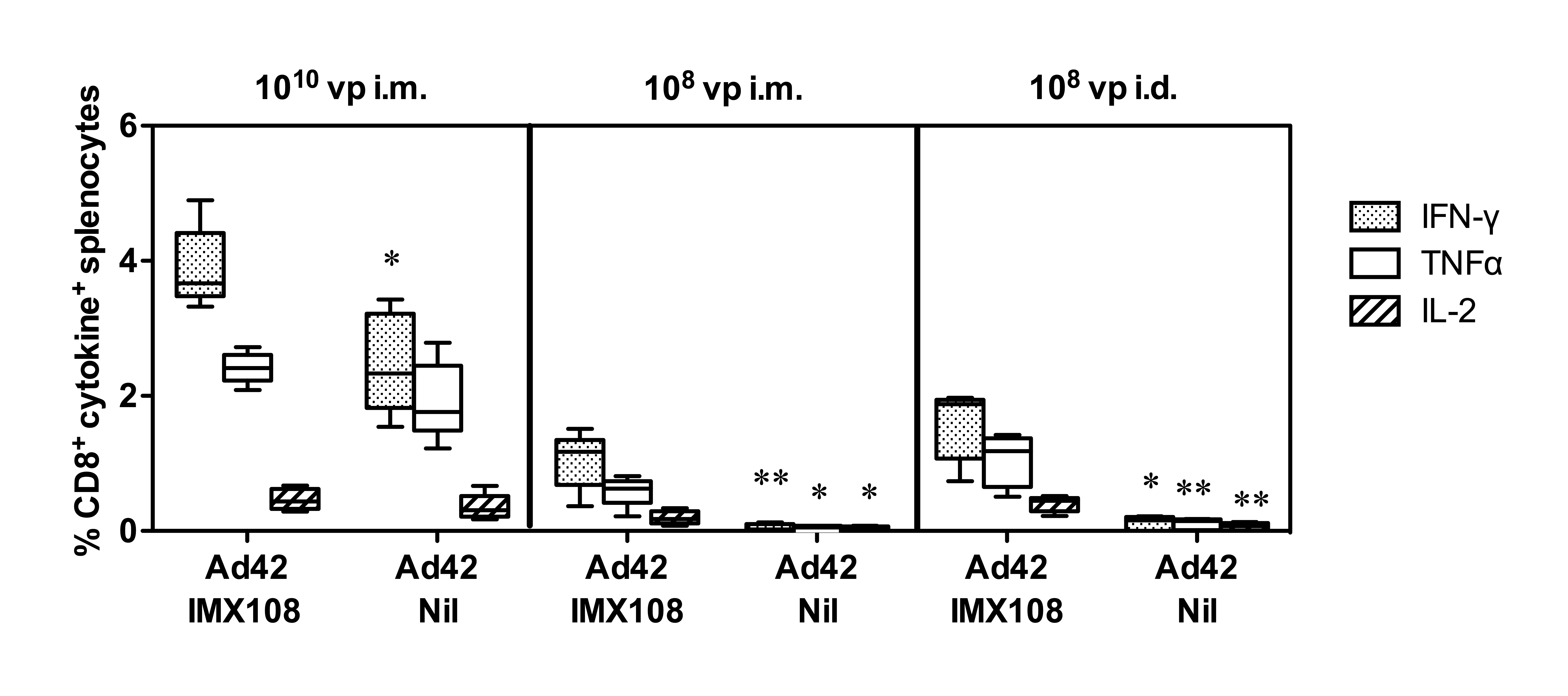

Supplement: Figure S1 — Adjuvant activity of IMX108 following AdHu5 vaccine dosing based on viral particles (vp). BALB/c mice were immunized with 1×10^10^ vp or 1×10^8^ vp Ad42-IMX108 or Ad42-Nil by either the i.m. or i.d. route as indicated. Two weeks later frequency of PyMSP133-specific IFN-γ, TNFα and IL-2 positive CD8+ splenic T cells was measured by ICS. Box and whisker plots show median, IQR and range for n = 5 mice/group. **P<0.01, *P<0.05 by Mann Whitney test compared to the Ad42-IMX108 given by the same dose and route. (TIFF) [file pone.0044943.s001.tiff]

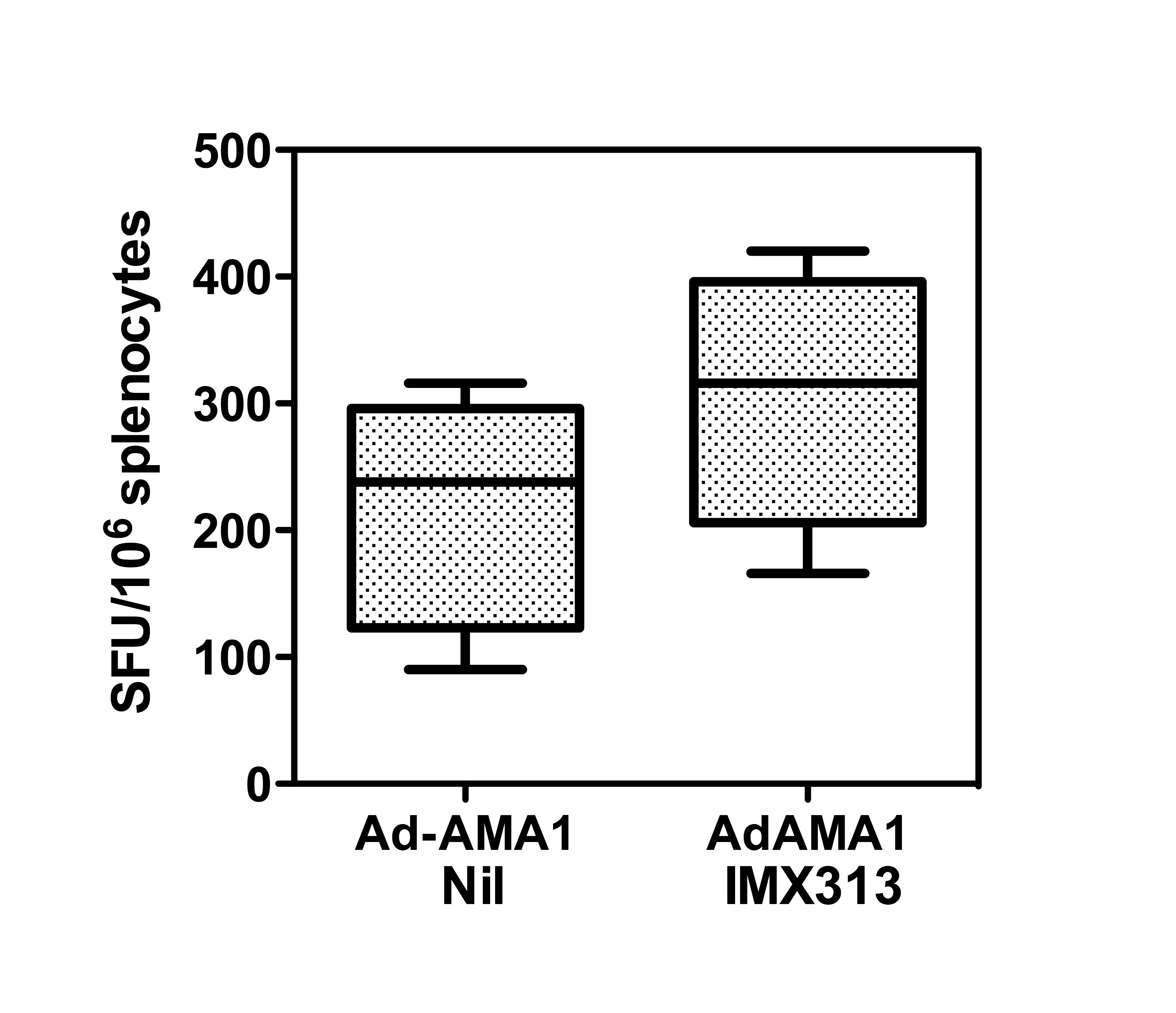

Supplement: Figure S3 — Adjuvant activity of IMX313 following Ad-AMA1 immunisation assessed by IFN-γ ELISPOT. BALB/c mice were immunized i.m. with 1×10^7^ ifu AdHu5-PfAMA1 with or without IMX313 (AdAMA1-IMX313 and AdAMA1-Nil). Fourteen days later mice were culled and AMA1-specific IFN-γ positive splenocytes were measured by ELISPOT. Data shown are spot forming units (SFU) per 106 splenocytes (n = 5 mice per group). Box and whisker plots indicate median, IQR and range. (TIFF) [file pone.0044943.s003.tiff]
